# Supplementary material for: Atrophin controls developmental signaling pathways via interactions with Trithorax-like
Source: eLife. 2017 Mar 22;6:e23084. doi: 10.7554/eLife.23084 (PMC5409829; doi:10.7554/eLife.23084)
Supplement: Supplementary file 3. — DOI: http://dx.doi.org/10.7554/eLife.23084.028 [file elife-23084-supp3.docx]

**Sample size**

Main Figures

Figure 2

A: >10 different biological replicates of antennal discs were stained and analyzed. >10 clones were analyzed.

B, C: >10 different biological replicates of wing discs were stained and analyzed. >10 clones were analyzed.

Figure 3

B: 4 independent *in situ* hybridization experiments, each time involving ≥10 wing discs, were analyzed.

C-E: >10 different biological replicates of wing discs were stained and analyzed. >10 clones were analyzed.

Figure 4

C: >5 different biological replicates of wing discs were stained and analyzed. >10 clones were analyzed.

D, E: >10 different biological replicates of eye discs were stained and analyzed. >10 clones were analyzed.

Figure 6

D, F: 4 independent experiments were performed to generate and stain *Trl^R85^* clones each. ≥10 wing discs were stained each time

Figure Supplement

Figure 4 – Figure Supplement 1

A: >5 different biological replicates of wing discs were stained and analyzed. >10 clones were analyzed.

B: >10 different biological replicates of antennal discs were stained and analyzed. >10 clones were analyzed.

C: 4 independent in situ hybridization experiments, each time involving ≥10 wing discs, were analyzed.

Figure 4 – Figure Supplement 2

A-B: >5 different biological replicates of eye discs were stained and analyzed. >10 clones were analyzed.

Figure 4 – Figure Supplement 3

A-C: >10 different biological replicates of eye discs were stained and analyzed. >10 clones were analyzed.

Figure 6 – Figure Supplement 3

4 independent experiments were performed to generate and stain *Trl^R85^* clones each. ≥10 wing discs were stained each time.
